# Von Willebrand Factor Facilitates Intravascular Dissemination of Microsporidia *Encephalitozoon hellem*

**DOI:** 10.3389/fcimb.2021.694957

**Published:** 2021-05-21

**Authors:** Jialing Bao, Biying Mo, Guozhen An, Jian Luo, Mortimer Poncz, Guoqing Pan, Tian Li, Zeyang Zhou

**Affiliations:** ^1^ State Key Laboratory of Silkworm Genome Biology, Southwest University, Chongqing, China; ^2^ Chongqing Key Laboratory of Microsporidia Infection and Control, Southwest University, Chongqing, China; ^3^ Department of Pediatrics, The Perelman School of Medicine, University of Pennsylvania, Philadelphia, PA, United States; ^4^ College of Life Sciences, Chongqing Normal University, Chongqing, China

**Keywords:** von Willebrand factor, *Encephalitozoon hellem*, microsporidia, intravascular dissemination, infection

## Abstract

Microsporidia are a group of spore-forming, fungus-related pathogens that can infect both invertebrates and vertebrates including humans. The primary infection site is usually digestive tract, but systemic infections occur as well and cause damages to organs such as lung, brain, and liver. The systemic spread of microsporidia may be intravascular, requiring attachment and colonization in the presence of shear stress. Von Willebrand Factor (VWF) is a large multimeric intravascular protein and the key attachment sites for platelets and coagulation factors. Here in this study, we investigated the interactions between VWF and microsporidia *Encephalitozoon hellem* (*E. hellem*), and the modulating effects on *E. hellem* after VWF binding. Microfluidic assays showed that *E. hellem* binds to ultra-large VWF strings under shear stress. *In vitro* germination assay and infection assay proved that *E. hellem* significantly increased the rates of germination and infection, and these effects would be reversed by VWF blocking antibody. Mass spectrometry analysis further revealed that VWF-incubation altered various aspects of *E. hellem* including metabolic activity, levels of structural molecules, and protein maturation. Our findings demonstrated that VWF can bind microsporidia in circulation, and modulate its pathogenicity, including promoting germination and infection rate. VWF facilitates microsporidia intravascular spreading and systemic infection.

## Introduction

Microsporidia are a group of intracellular parasites that have recently been re-classified to fungi (Hirt et al., 1999; Han and Weiss, 2017). The host range of microsporidia is extremely wide, and at least 15 species are human pathogens with the major ones being *Enterocytozoon bieneusi* (*E. bieneusi*), *Encephalitozoon hellem* (*E. hellem*), *Encephalitozoon cuniculi* (*E. cuniculi*) and *Encephalitozoon intestinalis* (*E. intestinalis*) (Weiss, 2001; Valencakova and Danisova, 2019). Microsporidia extrude the polar tube inside-out to inject sporoplasm into the host cells. This process is called germination and is the key step for infection (Franzen, 2005). Inside the host cell, the sporoplasm proliferates and form more new spores that will further infect surrounding cells (Weber et al., 1993; Meissner et al., 2012). Microsporidia infections could be local and restrained, yet systemic even fatal infections are not rare (Weber et al., 1994; Weiss, 1995; Meissner et al., 2012). Microsporidia spores may disseminate systemically *via* intravascular system (Anderson et al., 2019; Han et al., 2019), however the mechanistic details of dissemination *via* circulatory system have not been fully examined.

In circulatory system, Von Willebrand factor (VWF) mediates the binding and activation of various cells and molecules such as platelets and factor VIII (Sadler, 1998; Yee et al., 2014; Lenting et al., 2015; Dong et al., 2019). Furthermore, the involvements of VWF in pathogen dissemination and inflammation have been reported in multiple settings. During acute infections, such as *Escherichia coli* infection may induce the haemolytic uremic syndrome, triggering the formation of microvascular thrombi mediated by Von Willebrand Factor (VWF) (Zheng and Sadler, 2008; Pillai et al., 2016; Ueda et al., 2017). Studies also revealed that VWF is able to directly bind to *Staphylococcus aureus* in blood under shear stress and promote intravascular infection of the sub-endothelium (Viela et al., 2019). VWF is also found to bind *Streptococcus pneumonia*, promoting pathogen aggregation and attachment to the endothelium surface (Jagau et al., 2019; Viela et al., 2019). In addition, malarial parasitemia caused by *Plasmodium vivax* also involves VWF binding and endothelial activation (Barber et al., 2015). Furthermore in chronic infection conditions, the endothelium damage and related plasma VWF levels increasement are reported. These conditions include carcinomas, chronic parasites infections and human immunodeficiency virus (HIV) infections (Park et al., 2012; van den Dries et al., 2015; Kong et al., 2020), and those individuals are susceptible groups of microsporidia infections.

VWF is a large multidomain protein. The type D domain (VWFD) in D’D3 assembly is not only essential for factor VIII binding but also crucial for multimerization of VWF (Dong et al., 2019). More importantly, VWFD domain is highly conserved in a lot of proteins such as vitellogenin and mucins, and these proteins have been reported to be mediators of pathogen invasion and dissemination in hosts (Sicard et al., 2017; Meng et al., 2018). Based on above facts, it is of great interest to investigate the essential role of VWF in mediating microsporidia dissemination and systemic infections *via* circulatory system.

Here in this study, we used the microsporidia *E. hellem* as a representative infection agent. We utilized various *in vitro* and *in vivo* methods to investigate the interactions between *E. hellem* and VWF. We proved that *E. hellem* spores could directly bind to VWF multimers under shear stress, and the D’D3 domain is essential for the direct interaction. Upon VWF binding, the germination and infection rates of *E. hellem* were significantly increased. Mass spectrometry analysis revealed various biological processes, such as metabolic activities, increased levels of structure molecule levels, and protein maturation of *E. hellem* were affected by VWF interaction. Together, our study is the first to describe key roles of VWF in microsporidia hematogenous dissemination.

## Materials and Methods

### VWF Proteins

Native full-length human VWF, termed FL-VWF, was purchased from Abcam (ab88533, Abcam, USA). Recombinant VWF containing VWFD domain in the partial-length D’D3 assembly (S764-C1130, His-tagged), was expressed and purified from Rosetta (DE3) cells transformed with His-tagged pET32 plasmid (Novagen) containing the target sequence (Robertson et al., 2008). The partial length of D’D3 assembly excluded several cysteines that are essential for disulfide bonding, aiming for better solution of the expressed protein. Yet the recombinant protein was retained in the inclusion bodies thus dissolved in 8 M Urea, 20 mM Tris–Hcl, 0.5 M NaCl, 1mM DTT, 1mM 2-mercaptoethanol at pH 8.0, and then filtered and loaded onto HiTrap™ chelating column (GE Healthcare Life Sciences, USA). Refolding of the bound proteins is achieved by very slowly (0.1 ml/min) wash the column with a liner 8-0M urea gradient, and then eluted by imidazole-containing elution buffer (Duan et al., 2006; Volonte et al., 2011).

### *E. hellem* Microsporidia

*E. hellem* strain (ATCC 50504/50451) was a gift from Professor Louis Weiss (Albert Einstein College of Medicine, USA). Rabbit kidney cells (RK13, ATCC CCL-37) were cultured in 10% fetal bovine serum (FBS, ThermoFisher) containing Minimum Essential Medium Eagle (MEM, Gibco) with penicillin (100 U/ml)–streptomycin (100 µg/ml) at 5% CO_2_. Confluent monolayers were infected with *E. hellem*. The spores were collected from culture media, purified by passing them through a 5 μm size filter (Millipore=) to remove host cells, concentrated by centrifugation, and stored in sterile distilled water at 4°C (Visvesvara et al., 1991). Spores used in these experiments were counted with a hemocytometer (three times/sample) and averaged.

### Microfluidic Chamber VWF Binding Assay

FL-VWF protein (20 µg/ml) was perfused through a flow chamber slide (µ-slide I luer, Cat# 80176, Ibidi, Germany), with shear stress of 5 dyn/cm^2^ for 2 min with the same concentration of bovine serum albumin (BSA) (Sangon Biotech) used as a control. *E. hellem* spores (10^5^/ml) were then perfused through the channel for 1 min. The channels were washed with PBS, and then fixed with 4% paraformaldehyde. The VWF “strings” along the channel were visualized under a fluorescent microscope after incubation with anti-VWF IgG (ab6994, Abcam, USA) followed by Alexa 594-labeled secondary antibody. The *E. hellem* spores were visualized by Calcofluor-white (CFW) (Sigma-Aldrich), a specific dye for chitin on the microsporidia spore surface (Luna et al., 1995).

### Recombinant VWF-D’D3 Assembly Binding to *E. hellem* Spore

Recombinant VWF-D’D3 (partial length, containing VWFD) (20 µg/ml) was incubated with *E. hellem* spores (10^7^/ml) for 30 min, and then the spores were washed and fixed. The control group was incubated with the same concentration of EGFP (Enhanced Green Fluorescent Protein), also expressed, expressed and purified from *EGFP*-containing pET32 transformed DE3 cells. Direct interaction between VWF-D’D3 and *E. hellem* was observed by fluorescent microscope using anti-VWF IgG (ab6994, Abcam) followed by Alexa 488-labeled secondary antibody, and DAPI (4’,6-diamidino-2-phenylindole) (Sigma-Aldrich), respectively.

To further investigate the binding specificity, microfluidic chamber assay was applied. FL-VWF protein (20 µg/ml) was perfused through a flow chamber slide (µ-slide I luer, Cat# 80176, Ibidi, Germany), with shear stress of 5 dyn/cm^2^ for 2 min. Next, recombinant VWF-D’D3 (20 µg/ml) pre-incubated *E. hellem* spores (10^5^/ml) or same concentration of EGFP protein pre-incubated *E. hellem* spores (10^5^/ml) were perfused through the chamber for 1 min. The channels were washed with PBS, and then fixed with 4% paraformaldehyde. The VWF “strings” along the channel were visualized under a fluorescent microscope after incubation with anti-VWF IgG (ab6994, Abcam, USA) followed by Alexa 594–labeled secondary antibody. The *E. hellem* spores were visualized by Calcofluor-white (CFW) (Sigma-Aldrich), and the recombinant VWF-D’D3 was visualized by anti-His antibody (SAB1305538, Sigma-Aldrich, Canada) followed by Alexa 488-labeled secondary antibody.

### *E. hellem* Germination and Infection

Untreated or pre-incubated *E. hellem* spores were subjected to germination, triggered by germination buffer (140 mM NaCl, 5 mM KCl, 1 mM CaCl_2_, 1 mM MgCl_2_, pH 9.5) at 37°C for 10 min, and then 5% (v/v, final ratio) H_2_O_2_ (Sangon Biotech) was added for 5 min (Leitch et al., 1993; He et al., 1996; Pattana Jaroenlak et al., 2020).

For infection assay, human foreskin fibroblast cells (HFF, ATCC CRL-2522) were maintained in Dulbecco’s Modified Eagle Medium (DMEM, ThermoFisher Scientific) with penicillin-streptomycin (ThermoFisher Scientific) supplemented with 10% FBS (ThermoFisher Scientific) at 5% CO_2_. The *E. hellem* spores were then added to HFF cells (20:1 spores/cells) and co-culture for various time periods. The infection rate of *E. hellem* was assessed by FISH (fluorescence *in situ* hybridization) assay, using Cy3-labeled oligonucleotide probes targeted to species-specific sequences of *E. hellem* 16S rRNA (5’-ACTCTCACACTCACTTCAG-3’) to specifically label the proliferating *E. hellem* inside host cells. In brief, *E. hellem* infected HFF cells were fixed, then incubated with hybridization buffer (900 mM NaCl, 20 mM Tris pH 7.5, 0.01% SDS) at 46°C for 12 h. Intracellular *E. hellem* in the host cells was visualized using fluorescently labeled probe (5 pM) under microscopy. The host cells were visualized by DAPI staining. The infection rate was calculated by the ratio of FISH-positive HFF cells over all cells in 20 randomly selected fields.

### Label-Free Quantitative Mass Spectrometry

Freshly purified *E. hellem* spores (10^8^/ml) were incubated with FL-VWF (20 µg/ml) for 30 min. The spores were then washed with PBS. To extract the total protein, experimental and control spores not exposed to VWF were lysed with 1 ml of SDT-lysis buffer (4% SDS, 0.1 mol/l dithiothreitol, and 0.1 mol/l Tris HCl, pH 7.6) with 10 μl Protease Inhibitor Cocktail (Sangon Biotech) using acid-washed glass beads (diameter: 425–600 µ, Sigma) in a Precellys-24 (Bertin Technologies). Triplicate protein samples were prepared from each experiment, and three experiments were performed. The samples were then subjected to label-free quantitative mass spectrometry.

### Statistics

Results of the *E. hellem* germination and infection ratios were compared using paired Student’s t-test. Statistical analysis of the mass spectrometry results were conducted using a one-way ANOVA followed by Bonferroni’s post-hoc test was used to show significant differences in protein expression. Statistical significances were analyzed and represented with F values, degree of freedom, as well as with P values.

## Results

### *E. hellem* Binds to Ultra-Large VWF Under Shear Stress

**Figure 1:**
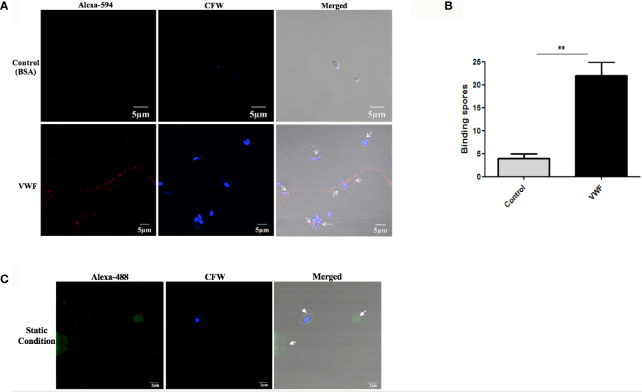
*E. hellem* spores attach to FL-VWF under flow. **(A)** Representative images of control protein (BSA, top) or FL-VWF (bottom), both at 20 µg/ml were perfused through the microfluidic chamber with *E. hellem* spores (10^5^ cells/ml) under flow at 5 dyn/cm^2^, respectively. The channels were then washed, fixed and stained by Alexa-594-labeled anti-VWF antibody and calcofluor-white (CFW). The fluorescent microscopy analysis showed that the VWF formed ultra large multimers under flow (red), and *E. hellem* spores (blue) attached to the strings of ultra large VWF strings, as pointed out by white arrows in the right figures. (Scale bar = 5 µm). **(B)** The number of binding spores in the channels were calculated, based on three independent studies with 8 random fields for each study (F(1,23) = 2.25, **P <0.01). **(C)** Under static conditions with no shear, the FL-VWF clumped and aggregated together (green). The *E. hellem* spores (blue) are not able to bind to clumped VWF.

To investigate whether VWF is essential for hematogenous dissemination of microsporidia, FL-VWF was perfused with *E. hellem* spores through the microfluidic chamber under shear stress. After washing and fixation, VWF strings and *E. hellem* spores were visualized by fluorescent microscopy. As shown in **Figures 1A,B**, *E. hellem* spores specifically attached to the VWF oligomers under shear stress, while no binding to control protein BSA. Also, the shear stress is important for *E. hellem* binding on VWF, for VWF undergoes a conformational transition from a compacted, globular to an extended form (Vergauwe et al., 2014). The inference is proved in **Figure 1C**, showing that when no shear stress presents VWF clumped together and no *E. hellem* binding on it. These results further confirmed the importance of VWF mediating microsporidia dissemination under physiological conditions.

### The VWF-D’D3 Assembly Is Key Binding Region for *E. hellem* on VWF

**Figure 2:**
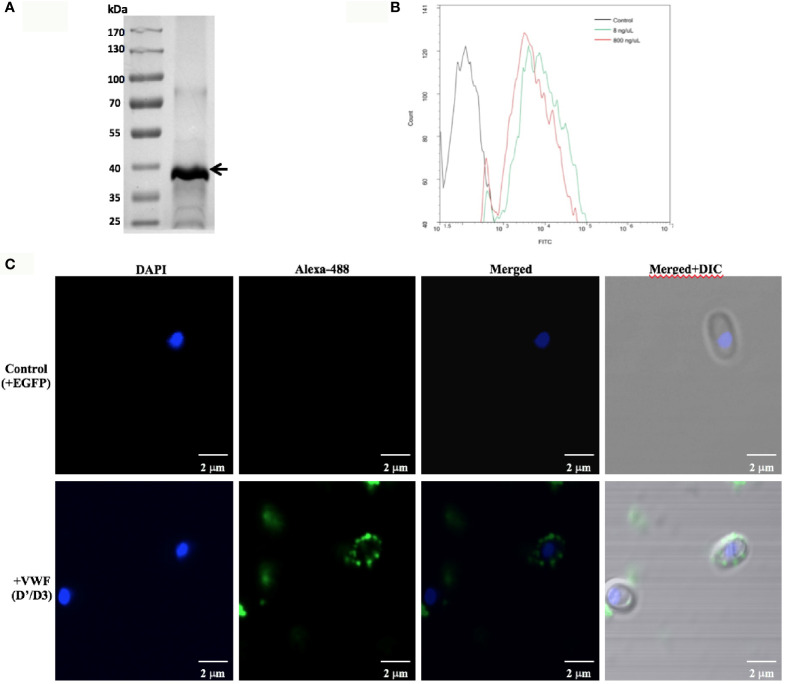
VWF-D’D3 binds to *E. hellem spores*. **(A)** Coomassie staining of recombinant VWF-D’D3. Arrow shows the major protein size at the expected size ~40 kDa. **(B)** Flow cytometry analysis of D’D3 binding to *E. hellem*. *E. hellem* spores (1 × 104 cells) were incubated respectively with, isotype antibody control (in black line), 8 ng/µl recombinant VWF-D’ D3 (in green line), and 800 ng/µl recombinant VWF-D’D3 (in red line). The result showed that with the increasing amount of recombinant VWF adding, the fluorescence signal increased as well. **(C)** Representative images of *E. hellem* spores were incubated with either control, recombinant EGFP (top) or VWF-D’D3 (bottom), both at 20 µg/ml for 30 min, then the spores were washed by PBS. After fixation, the direct interaction between VWF (green) and *E. hellem* (blue) was observed by fluorescent microscope (Scale bar = 2 µm).

Next, we investigated whether the VWFD domain containing D’D3 assembly is key binding region for *E. hellem*. The purified recombinant VWF-D’D3 assembly (**Figure 2A**) was incubated with *E. hellem* spores, and the binding effect was proved by flow cytometry and fluorescent microscopy analysis (**Figures 2B,C**).

**Figure 3:**
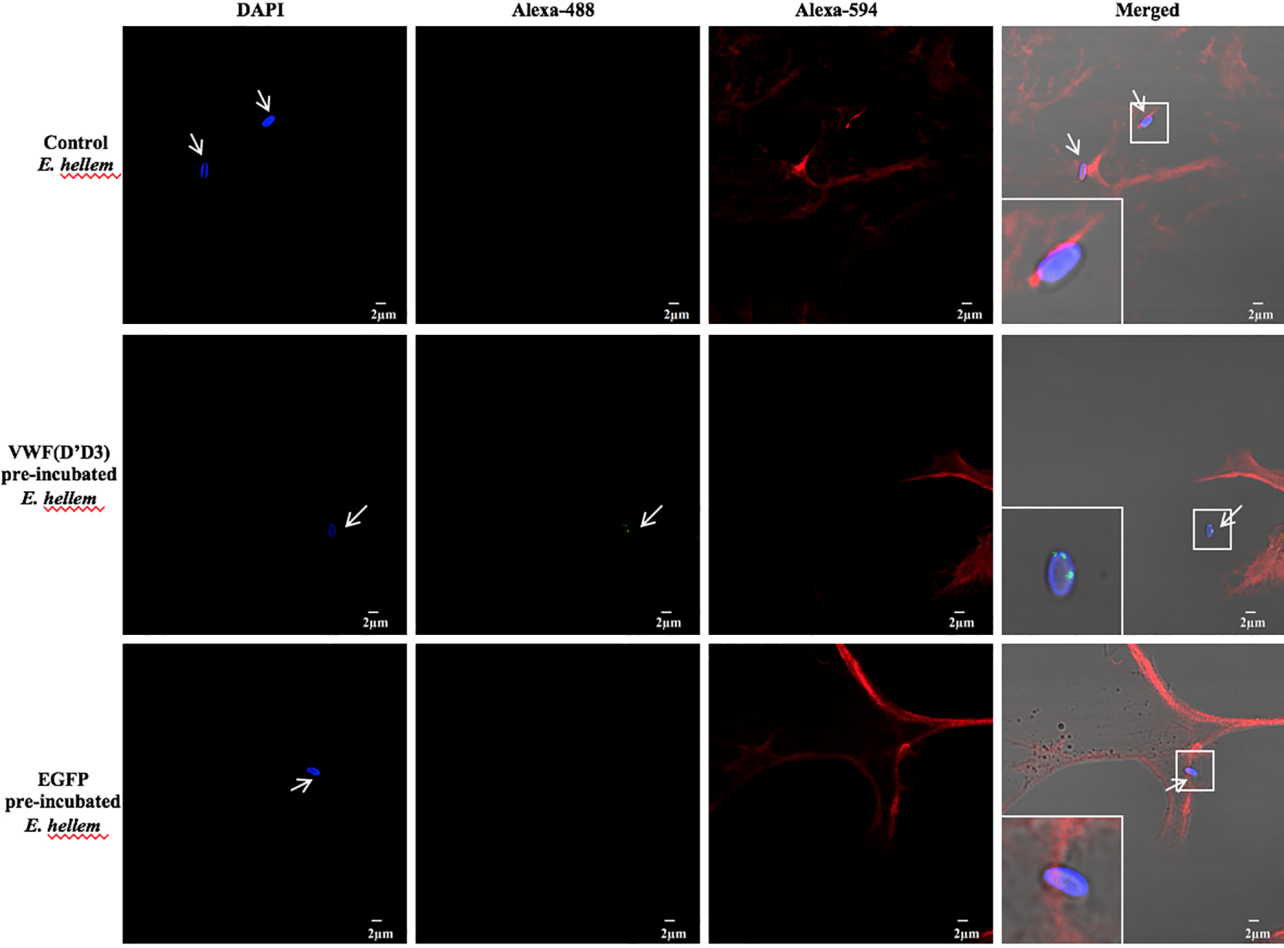
VWF-D’D3 is key binding region for *E. hellem*. In microfluidic chamber, full length VWF (20 µg/ml) was perfused with shear stress of 5 dyn/cm^2^ for 2 min. Same concentration (10^5^/ml) of either control (un-treated) *E. hellem* spores, VWF-D’D3 pre-incubated *E. hellem* spores, or EGFP pre-incubated *E. hellem* spores were then perfused through. The channels were then washed and fixed. The *E. hellem* spores were visualized by DAPI (blue), and the pre-incubated VWF-D’D3 which has attached to *E. hellem* spores were visualized by anti-His antibody followed by Alexa 488-labeled secondary antibody (green). The VWF oligomers were visualized by anti-VWF antibody followed by Alexa 594-labeled secondary antibody (red). As shown by this immunofluorescence assay, untreated *E. hellem* spores or un-related EGFP treated *E. hellem* spores were both able to attach to the VWF oligomer strings (arrows, and also shown in enlarged views in upper and bottom rows). While VWF-D’D3 pre-incubation occupy the binding site of *E. hellem*, thus the spores could not bind with VWF strings (arrow, and also shown in enlarged view in middle row).

To further confirm the key role of D’D3 assembly in *E. hellem*-VWF binding, recombinant D’D3 assembly was applied to pre-incubate with *E. hellem* and then the spores were perfused with FL-VWF in microfluidic chamber under shear stress. As shown in **Figure 3** that, D’D3 pre-incubation interferes with *E. hellem*-VWF binding while pre-incubation with un-related protein EGFP had no interference effect. These results indicated that the binding site was pre-occupied by the assembly, and D’D3 assembly is the key binding region for *E. hellem* interaction on VWF.

### VWF Binding Promotes *E. hellem* Germination

**Figure 4:**
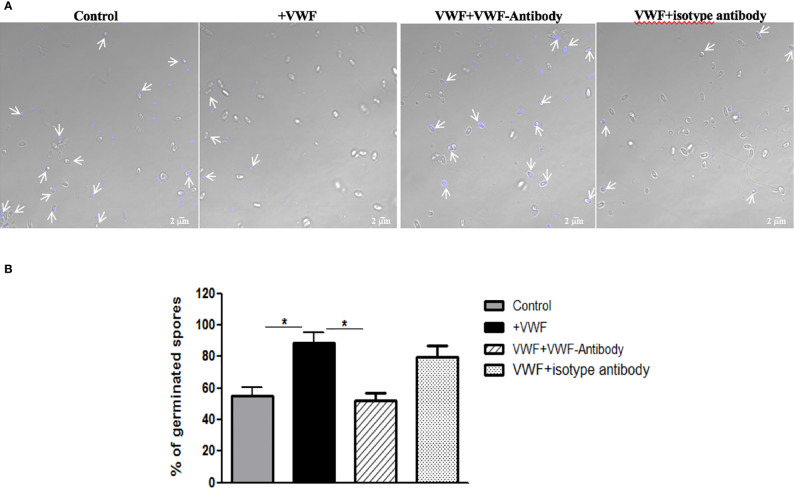
VWF binding promotes *E. hellem* germination. **(A)** Representative images of *E. hellem* germination affected by VWF. The control group *E. hellem* spores were untreated by any protien; The VWF group *E. hellem* spores were incubated with FL-VWF; The VWF + VWF-antibody group *E. hellem* spores were treated by VWF together with anti-VWF antibody; The VWF + isotype antibody group *E. hellem* spores were treated by VWF together with isotype antibody control. All the spores from each group were then stimulated with germination buffer to further trigger germination. The *E. hellem* spores were then stained by DAPI, and un-germinated spores will show blue color (pointed out by arrows). (Scale bar = 5 µm). **(B)** Germination rates were calculated by the ratio of germinated spores over all spores, based on three independent studies with 10 random fields per study. The results showed that VWF treatment significantly promoted *E. hellem* spores’ germination (F(1,29) = 1.89, *P <0.05), and this effect was inhibited by VWF specific antibody (F(1, 29) = 2.09, *P <0.05).

We next examined whether binding to VWF by microsporidia would influence the biology and potentially influence systemic infection by this organism. We first examined whether binding of spores to VWF influences germination. Freshly purified *E. hellem* spores (10^8^/ml) were incubated with FL-VWF for 1 h. Controls were either untreated *E. hellem* spores, spores incubated with VWF together with a blocking anti-human VWF antibody (Abcam, USA), or spores incubated with VWF together with an isotype antibody control. After incubation, *E. hellem* spores from each group were washed with PBS and then subjected to germination accordingly. Under fluorescent microscope, untreated *E. hellem* spores will show blue color due to DAPI staining of their nuclei; while germinated spore will show no color as the sporoplasms with their nuclei had already been extruded. The germination rate was then assessed by calculating the ratio of germinated spores over all spores under the view. Results showed that incubation of the spores with VWF significantly promoted *E. hellem* germination, and this effect was inhibited specifically by blocking anti-VWF antibody (**Figure 4**).

### VWF-Bound *E. hellem* Demonstrates Enhanced Host Cell Infectivity

**Figure 5:**
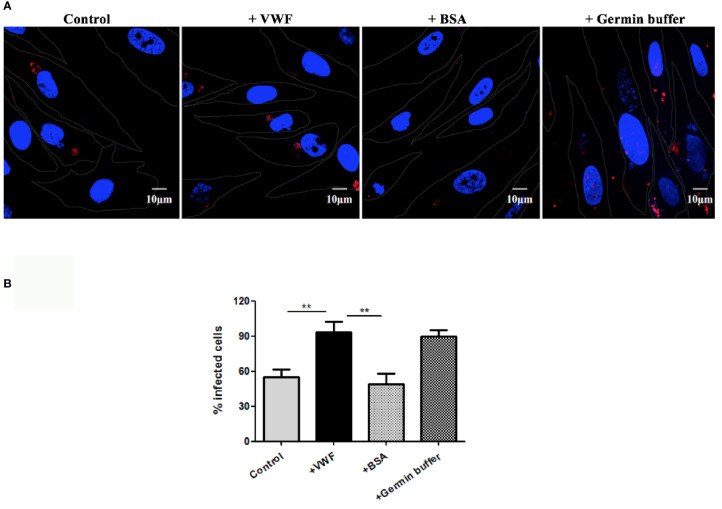
VWF promotes *E. hellem* infection. **(A)** Representative fields of HFF cells exposed to *E. hellem* spores that had undergone no treatment (control) or binding of either FL-VWF or BSA or exposed to germination buffer. The spores were then added to HFF cells and culture for 12 h. The HFF cells outlines were depicted as ‘dots’ by Adobe IIIustrator CS6to the DIC images of the cells. HFF cell nuclei were stained by DAPI (blue), while the infected *E. hellem* was represented by FISH probe (red) (Scale bar = 10 µm). **(B)** Infectivity rate was the ratio of infected HFF cells over all cells, based on three independent studies with 20 random fields per study. The result showed that VWF treatment significantly increased the infection rate of *E. hellem* to host cells (F(1,59) = 2.42,**=P <0.01); while the un-related protein treatment of *E. hellem* spores had no effect on the infection ability (F(1,59) = 1.92, **P <0.01).

Another potential manner by which VWF may enhance systemic spread of microsporidia infection is by enhancing its ability to infect host cells. We examine this issue by pre-incubating *E. hellem* spores with FL-VWF, while the controls were either untreated *E. hellem* spores or spores treated with BSA. Another control was to pre-germinate the spores to enhance infectivity. The various pre-treated *E. hellem* spores were then co-cultured with HFF cells to allow infection, and then washed and fixed. The proliferating *E. hellem* inside the host cells were visualized by fluorescently labeled FISH probe. The infection rate was calculated by the ratio of FISH-positive HFF cells over total HFF cells. As shown in **Figure 5**, the infectivity of *E. hellem* was significantly increased after FL-VWF incubation, almost to the level of pre-germinated spores.

### Mass Spectrometry Analysis of the Impacts of VWF Binding on *E. hellem*

**Figure 6:**
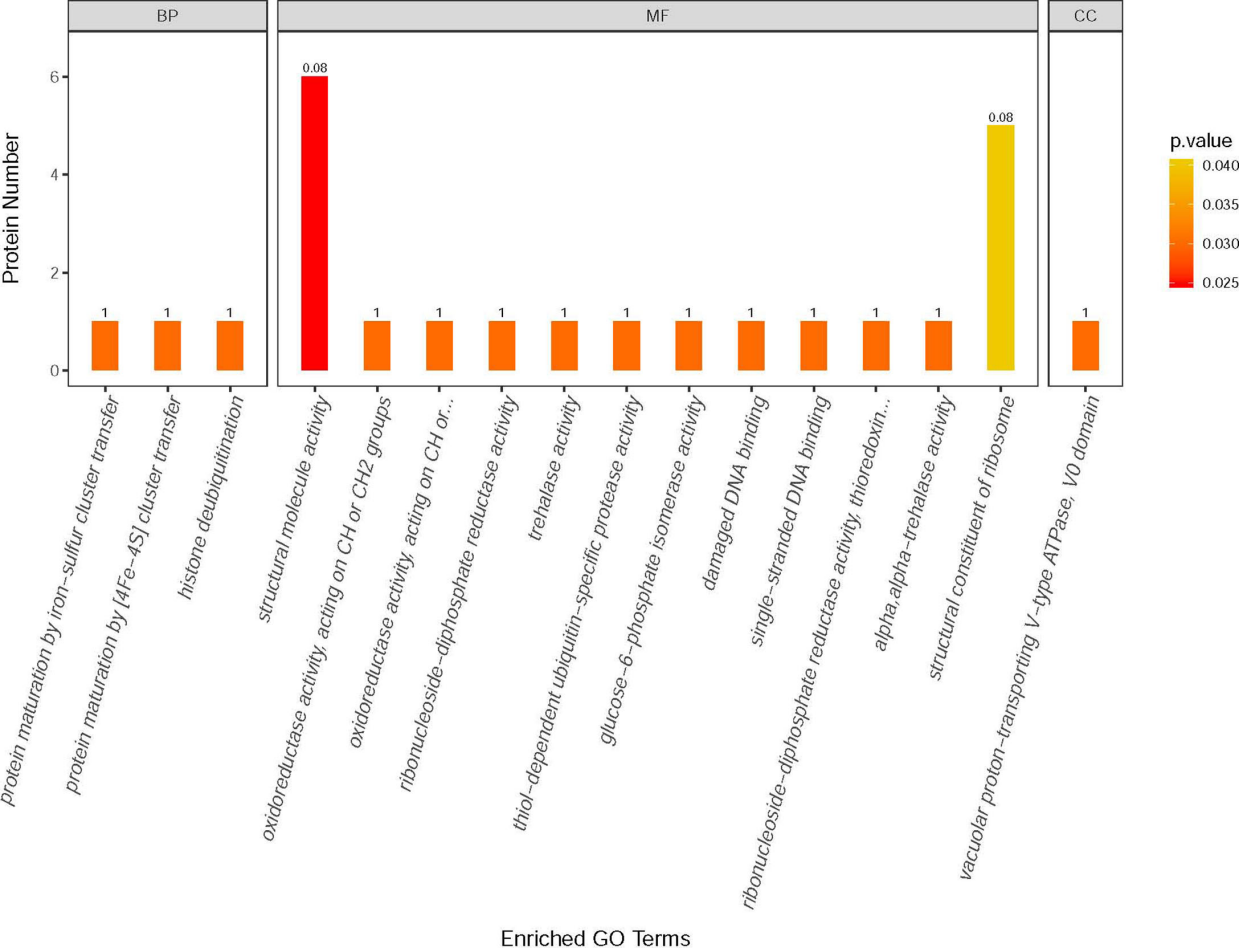
GO annotation and enrichment analysis of differentially expressed proteins in *E. hellem* post-exposure to VWF. The primary Y axis denotes the number of annotated proteins categorized to each GO term. The secondary Y axis represents the percentage of annotated proteins to each GO term in all differential proteins. GO terms are classified into three subcategories, including biological process (BP), molecular function (MF) and cellular compartment (CC). The color gradient represents the p-value; the closer to red, the smaller the p-value. The enriched proteins are categorized and showed on X axis as: 1—Protein maturation by iron-sulfur cluster transfer; 2—Protein maturation by [4Fe–4S] cluster transfer; 3—Histone deubiquitination; 4—Structural molecule activity; 5—Oxidoreductase activity; 6—Oxidoreductase activity, acting on CH2 groups; 7—Ribonucleoside-diphosphate reductase activity; 8—Trehalase activity; 9—Thiol-dependent ubiquitin-specific protease activity; 10—Glucose-6-phosphate isomerise activity; 11—Damage DNA binding; 12—Single-stranded DNA binding; 13—Thioredoxin activity; 14—Alpha trehalase activity; 15—Structural constituent of ribosome; 16—Vacuolar proton-transporting V-type ATPase.

Label-free quantitative mass spectrometry was utilized to analyze the *E. hellem* protein change after VWF incubation. Various proteins were significantly increased, including ones involved in metabolic activities, DNA synthesis and intracellular transportation. Changes in the levels of specific proteins of either an increase or a decrease of two fold following FL-VWF binding are shown in **Table 1**. The differentially expressed proteins were further subjected to gene ontology (GO) annotation and enrichment analysis, as shown in **Figure 6**. Various aspects of *E. hellem* are altered after VWF binding, including biological process, molecular function and cellular compartment.

**Table 1:** Representatives of ddifferentially expressed proteins of *E. hellem* after VWF incubation.

UniProtKB ID	Protein Name	Unique Peptides	Coverage	Up/Down-regulated
I6UNU1	Glucose-6-phosphate isomerase	55	69.8	Up
I6ULI4	40S ribosomal protein S6	14	38.2	Up
I6TLD3	Protein YOP1	10	33.5	Up
Q5VDH6	Aminopeptidase	2	52.7	Up
I6UEB3	HTH_9 domain containing protein	2	4.8	Up
I6TI03	Trehalase	19	31.2	Down
I6UNA0	Ribosomal protein L14E/L6E/L27E	10	54.7	Down
I6TWX8	V-type proton ATPase subunit a	8	12.1	Down
I6UP05	Translation initiation factor 2B subunit epsilon	3	6.3	Down
I6UM86	DNA polymerase sigma	1	5.4	Down

## Discussion

Current study is the first to show a direct interaction between plasma protein VWF and the microsporidia, *E. hellem*, and demonstrate that the binding of VWF to *E. hellem* spores significantly enhances their germination and infectivity abilities. Mass spectrometry analysis revealed that various proteins expression levels of *E. hellem* were altered after VWF interaction. For instance, glucose-6-phosphate isomerase, an enzyme involved in glucose metabolism (Kugler and Lakomek, 2000); YOP1, a protein associated with vesicle-mediated transportation and invasion (Viljanen et al., 1991); and aminopeptidase, an enzyme associated with parasitophorous vacuole formation (Lu et al., 2020), were all up-regulated. In the meantime, the translation initiation factor 2B, DNA polymerase, and trehalase, a protease responsible for metabolic process in extreme condition (Zhao et al., 2016), were all significantly down-regulated. These changes together indicate that binding by VWF signals *E. hellem* to slow-down regular DNA and protein synthesis, change the metabolism mode, accelerate vesicle transportation, and other modifications to prepare for germination by the pathogen and invasion of surrounding host cells.

VWF is an essential protein in coagulation and thrombosis, binding to platelet’s glycoprotein Ib/IX receptor, to circulating coagulation factor VIII and to exposed subendothelial collagen amongst other ligands (Sadler, 1998). It is known the D’D3 assembly of VWF is important for various ligands binding including coagulation factors FVIII, P-selectin and GpIba, and even some pathogens (Michaux et al., 2006; O’Seaghdha et al., 2006; Madabhushi et al., 2014; Yee et al., 2014). In particular, the D’ region (composed of TIL’ and E’ domains) is especially important for FVIII binding (Shiltagh et al., 2014). Thus in this study, we constructed the recombinant VWF-D’D3 contains full of TIL’, E’ and most part of D3 (S764-C1130). We aimed to have a construct which retains the full binding abilities but without the residues such as C1142 and C1222 for inter-chain disulfide bonding, so that will get homogenous monomeric protein (Hilbert et al., 2003; Shapiro et al., 2014; Lenting et al., 2015). With this protein, we managed to prove that D’D3 region is the key binding site for *E. hellem* on VWF, thus the occupation by *E. hellem* might interfere with physiologic functions of VWF and any related pathophysiologic processes. It would also be quite interesting to examine whether binding of microsporidia to the D’D3 region of VWF contributes to hemostatic conditions. A case study in a patient with acute myeloblastic leukemia who developed a systemic microsporidia infection also developed disseminate intravascular coagulopathy, consistent with VWF binding to microsporidia interfering with physiologic hemostatic (Yazar et al., 2003). Other reports also are consistent with systemic microsporidia effecting coagulation and thrombosis (Small et al., 2014; Bukreyeva et al., 2017; Pariyakanok et al., 2019). Bacterial binding to VWF promotes bacterial settlement, and facilitates the pathogens transmigration and into deeper tissue sites (Steinert et al., 2020). We hypothesized that binding of microsporidia to VWF may also be the underlying mechanism of local and disseminated inflammations. On the other hand, we would not exclude the role of phagocytotic cells facilitating microsporidia spreading, as doing so to other pathogens (Guirado et al., 2013; Delgado Betancourt et al., 2019). However, our preliminary data showed that microsporidia interaction with phagocytes down-regulated the cells’ maturation and proper functions such as migration abilities. Thus we hypothesized that microsporidia ‘spreading’ by the dysfunctional cells may not be as efficient as by shear stress in blood and by binding with VWF for better infection or transmigration to deeper tissues. Furthermore, considering the fact that VWF is a mediator for many other pathogens, such as *S. aureus* dissemination, it will be interesting and important to know whether the interaction with *E. hellem* interferes or facilitates co-infection with other pathogens.

The type D domain (VWFD) is not only presented in the VWF protein but also in many other proteins, such as mucin in the digestive tract (Bukreyeva et al., 2017). Considering the facts that the initial infection site for *E. hellem* may in the digestive tract, it would be important to investigate the interactions between *E. hellem* with those VWD domain containing proteins. In addition, vertical/transovarial transmission is a known feature of microsporidia, especially in invertebrates (Dunn et al., 2001). It has been shown that the VWD D’D3-like domain-containing protein vitellogenin has an essential role in vertical transmission and involves direct binding of pathogens at this domain (Raina et al., 1995; Herren et al., 2013). Thus, it is important to investigate whether VWF facilitates human infecting-microsporidia, such as *E. hellem*, to mediate transovarial transmission or assist in pathogen transmission *via* blood contamination during birthing (Kaneda et al., 1997; Murakami et al., 2012).

In conclusion, the present study revealed that VWF can directly bind the microsporidia *E. hellem*, at least in part, *via* its VWD domain. This interaction altered multiple biological aspects of the pathogen that eventually lead to enhanced germination and infectivity. These effects make VWF a candidate for being a key mediator of microsporidia intravascular dissemination, and provide insights into the mechanism(s) by which microsporidia can lead to endocarditis, thrombocytopenia and other systemic manifestations. There have no specific therapeutics for microsporidia. Drugs such as albendazole and fumagillin are either non-specific, not able to eliminate the pathogen, and have toxic side-effects (Didier et al., 2005). Thus, novel treatment strategies for microsporidia are necessary. Based on our findings, preventing the binding of microsporidia to VWF, probably *via* specific antibody neutralizing the binding site, may be an attractive target to prevent microsporidia dissemination and systemic infections.

## Data Availability Statement

The raw data supporting the conclusions of this article will be made available by the authors, without undue reservation.

## Author Contributions

JB designed the study and conducted most the experiments, interpreted the data, and wrote the manuscript. BM, GA, JL, TL and GP assisted in germination and infection experiments and analysis of data. MP contributed to study design and with ZZ contributed in manuscript grammar and language editing. All authors contributed to the article and approved the submitted version.

## Funding

This work is supported by Fundamental Research Funds for the Central Universities (No. XDJK2020B005) and The National Natural Science Foundation of China (No. 31802141).

## Conflict of Interest

The authors declare that the research was conducted in the absence of any commercial or financial relationships that could be construed as a potential conflict of interest.
